# Quantitative Analysis of Macular Retina Using Light Reflection Indices Derived from SD-OCT for Pituitary Adenoma

**DOI:** 10.1155/2020/8896114

**Published:** 2020-11-04

**Authors:** Min Sun, Hongming Zhang, Xinjian Chen, Qimi Zhang

**Affiliations:** ^1^Department of Electronic and Information Engineering, Wenzheng College of Soochow University, Suzhou, Jiangsu, China; ^2^Department of Electronic Engineering, Jiangsu Vocational College of Electronics and Information, Huaian, Jiangsu, China; ^3^School of Electronic and Information Engineering, Soochow University, Suzhou, Jiangsu, China

## Abstract

**Purpose:**

To quantitatively investigate the macular retinal light reflection characteristic using optical property indices derived from spectral-domain optical coherence tomography (SD-OCT) scans with depth attenuation compensation for pituitary adenoma.

**Methods:**

This study included 38 patients (mean age 44.66 ± 13.77 years old) with diagnosis of pituitary adenoma and 43 age-matched controls. All SD-OCT scans were light attenuation compensated by a depth-resolved model. Attenuation coefficient, the corrected intensity, and the retinal layer thickness were deduced for macular retinal nerve fiber layer (RNFL) and ganglion cell layer combined with inner plexiform layer (GCIPL), as well as comparing between patients and controls by statistical methods.

**Results:**

Attenuation coefficients of RNFL and GCIPL among patients were significantly lower compared to the controls with *P* values equal to or less than 0.001. The mean values of the corrected optical intensity were decreased in the patients without universally significant differences. Significant decreases in thickness existing in the RNFL of patients, especially in the superonasal (SN) quadrant and inferonasal (IN) quadrant (decrease ratio = 9.64% and 13.02%, both with *P* < 0.001). The thickness of RNFL was significantly associated with the attenuation coefficient (standardized beta = 0.335, *P*=0.002). The performances of attenuation coefficient were better than the corrected optical intensity and the thickness (the values of the areas under the receiver operating characteristic curves = 0.751 and 0.758, both with *P* < 0.001) in discriminating pituitary adenoma patients from controls.

**Conclusions:**

The retinal light reflection characteristics were debilitated in patients with pituitary adenoma. The potential of attenuation coefficients of RNFL and GCIPL in distinguishing patients with pituitary adenoma from controls was validated by the comparison of the derived optical property indices.

## 1. Introduction

Visual dysfunction induced by pituitary adenoma is ultimately attributed to the damage to retinal ganglion cells (RGC) through the compression or blood supply interference acting on the optic chiasm [1, 2]. Macula contains a large proportion of RGC neurons (about 34% of total macular volume) and glial cells [3]. Cross-sectional imaging of the macular retinal layers is increasingly used to reveal the axonal or neuronal damage to the retina in the field of visual function recovery prediction for pituitary tumors [4].

Optical coherence tomography (OCT) is widely used for retinal imaging to obtain information about the structural and pathophysiologic changes in the retinal layers. Image intensity based morphological and optical properties are the common focused issues in the retinal study. The morphological quantitative measurements reflect the thicknesses or shapes of the retinal layers. Light reflectivity of the retinal layer demonstrating different image optical intensities can be viewed as signs of tissue pathophysiologic changes, especially in intraretinal spaces, which sometimes do not show thickness changes [5, 6]. Using optical scattering properties can achieve reliable measurement of the changes involved in retinal pathological processes [6–8].

Contrast in OCT images originates from the differences in optical back-scattering or reflective properties of the retinal tissue [9, 10]. In consideration of the noise introduced due to the image quality and the photophysical processes that affect light propagation in retinal tissue, the OCT signal usually participates in retinal quantitative analysis in the form of alternative optical property parameters, such as attenuation coefficient [11, 12], rather than the raw OCT image intensity. The attenuation coefficient measures the power loss of a coherent light beam due to scattering and absorption during the propagation through a turbid medium and can be derived from the OCT image intensities using attenuation compensation models [12, 13].

In this study, we quantitatively investigated the retinal light reflection characteristics in the patients with pituitary adenomas using attenuation coefficient, the corrected intensity, and the retinal layer thickness derived from the spectral-domain OCT (SD-OCT) scans with depth attenuation compensation based on a single scattering model. The abilities to distinguish between the patients with pituitary adenomas and controls were also compared to the attenuation coefficient, the corrected intensity, and the retinal layer thickness.

## 2. Materials and Methods

### 2.1. Subjects

In this cross-sectional study, patients diagnosed with pituitary tumors and age-matched healthy people (controls) were recruited at the Nanjing General Hospital of Nanjing Military Command, China. The SD-OCT databases of all subjects were collected. This study was approved by the Institutional Review Board and adhered to the tenets of the Declaration of Helsinki. Informed consent was required from each subject with verbal permission during the process of OCT inspection. The main patient exclusion criteria were as follows: (1) presence of any optic disc anomaly or macular disease and (2) a history of ocular surgery. The control group consisted of healthy people with a normal ophthalmic examination.

### 2.2. Retinal Imaging and Preprocessing

All subjects underwent SD-OCT examination using the commercially available equipment Topcon DRI OCT-1 Atlantis (Topcon Corporation, Tokyo, Japan) with a center wavelength of 1050 nm. Macula centered OCT volumes were acquired for one eye using a standard 6 × 6 mm^2^ protocol, with 256 B-scan slices in each three-dimensional (3D) acquisition. The OCT image size was 992 × 512 × 256 voxels, with a resolution of 2.62 × 11.72 × 23.44 *μ*m^3^. The raw scanned data were exported from the OCT machine in .fds file format and were interpreted as 16-bit grayscale images resulting in 65,536 levels of gray expressed in arbitrary units (AU). The effectiveness of image analysis was ensured by eliminating defective images, such as those with eye movements and black bands throughout or other appearances that would impact the subsequent analysis.

**Figure 1:**
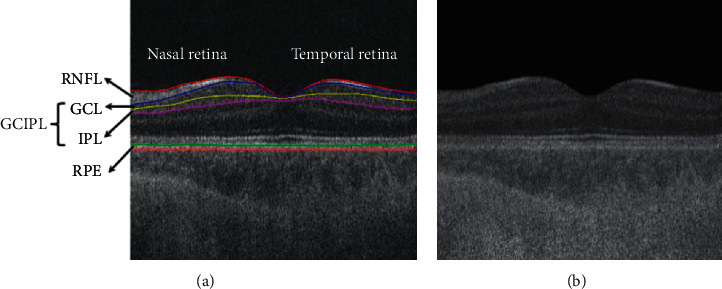
In vivo retinal scan of a normal subject. (a) Layer segmentation on the denoised and flattened image. (b) The attenuation coefficient image. RNFL: retinal nerve fiber layer; GCL: ganglion cell layer; IPL: inner plexiform layer; GCIPL: GCL combined with IPL; RPE: retinal pigment epithelium.

The SD-OCT data were firstly denoised by a speckle reduction method named enhanced low-rank + sparsity decomposition (EnLRpSD) [14]. We followed the methods of Sun et al. [11]; the retinal nerve fiber layer (RNFL), the ganglion cell layer (GCL), the inner plexiform layer (IPL) (GCIPL), and the retinal pigment epithelium (RPE) were automatically segmented using the 3D graph-based retinal layer segmentation approach applied on the SD-OCT data [15]. For the similar intensity presented in the OCT reflectivity profile, the GCL and IPL are usually analyzed together, that is, GCL combined with IPL form GCIPL. Each B-scan was flattened at Bruch's membrane to be convenient for quantitative analysis (Figure 1(a)). The segmentation results were reviewed by a retinal specialist, and the images with segmentation errors were excluded. For the local structural characteristic study, the macular area was intercepted by centring a square with a width of 5.0 mm at the fovea on the enface projection and was demarcated into four quadrants: superonasal (SN), inferonasal (IN), superotemporal (ST), and inferotemporal (IT). The thickness of each layer was measured by multiplying the resolution (2.6 *μ*m) by the total number of voxels between the top and bottom interfaces of the layer's *z-*axis direction. The resulting quadrantal thickness was the average of the thicknesses in each quadrant.

### 2.3. Optical Property Indices

To accurately study the light reflectivity property of the retina, the optical depth compensation of OCT signal is necessary. The denoised SD-OCT images were analyzed to quantify the depth-dependent attenuation coefficient *μ*(*z*) (expressed in *μm*^−1^, *z* expressed in *μm*) of the tissues by fitting the OCT signal to a depth-resolved single scattering model and discretizing [12, 13, 16, 17]. The attenuation corrected A-scan is deduced as

(1) $$\begin{array}{c} E_{R}\left(z\right)\approx E_{A}\left(z\right)\frac{\sum_{u=1}^{N}E_{A}\left(u\right)}{\sum_{u=z+1}^{N}E_{A}\left(u\right)}, \end{array}$$

where *E*_*R*_ (*z*) is the actual depth reflectivity profile, *E*_*A*_ (*z*) is the attenuated OCT A-scan signal, and *N* is the final pixel in the A-scan. Considering the noise floor and the depth-dependent sensitivity decay caused by the SD-OCT system [17], we determined the attenuation coefficients in practice to exclude the signal above the RNFL, which only consists of the noise, and to restrict the overcompensation in the deepest layer below the choroid and expressed them as

(2) $$\begin{array}{c} \mu \left(z\right)\approx \left\{\begin{array}{cc} 0,\; & \;z<z_{u-\text{RNFL}}\text{ and }z>z_{l-\text{choroid}},\\ \frac{E_{A}\left(z\right)}{2\sum_{u=z+1}^{N}E_{A}\left(u\right)},\; & z_{u-\text{RNFL}}\leq z\leq z_{l-\text{choroid}}. \end{array}\right. \end{array}$$

where *z*_*u*–RNFL_ and *z*_*l*–choroid_ refer to the upper boundary depth value of RNFL and lower boundary depth value of choroid in a single A-scan, respectively, *z*_*l*–choroid_ was obtained by extending the lower *z*–coordinate value of the retinal pigment epithelium (RPE) by 300 to cover the choroid area.  Figure 1(b) shows the attenuation coefficient image deduced from a macular SD-OCT B-scan of a healthy control. The attenuation calculation and the model implementation were done in Matlab release 2012a (Mathworks, Inc., Natick, MA, USA).

### 2.4. Statistical Analysis

The Shapiro–Wilk test was used to study the normality of the data. The data were compared between the patients and the controls by Mann–Whitney *U* test for nonnormal variables and unpaired *t*-test for normally distributed variables. Linear regression analysis was used to evaluate the association of the attenuation coefficient with the thickness and the corrected intensity. Receiver operating characteristic (ROC) analysis was used to assess the diagnostic performances of the indices. The Delong method was employed to evaluate the statistical significance of differences in the area under the ROC curve (AUC) values [18].

All statistical analyses were performed with the Statistical Package for Social Sciences (SPSS, version 22.0, IBM Corp., Armonk, NY) and MedCalc V.15.2 (Mariakerke, Belgium). *P* value less than 0.05 was considered statistically significant. The tables and plots in this paper were drawn by Excel (version 2013, Microsoft Corp., Redmond, WA), SPSS, and MedCalc.

## 3. Results and Discussion

### 3.1. Demographics

A total of 38 patients between 8 and 72 years of age and 43 normal controls between 23 and 71 years of age were recruited in this study. The patients had no significant age differences compared to the controls (44.66 ± 13.77 years old and 40.16 ± 12.49 years old, *P*=0.127), and neither group had significant gender differences with *P* value of 0.731. To avoid the correlation between both eyes of the same subject, only one eye of a subject was included.

### 3.2. Estimation of Indices

The mean and standard deviation of the thickness, the attenuation coefficient, and the corrected intensity in each quadrant were calculated by descriptive statistics for the macular RNFL and the GCIPL for all subjects (Table 1).

**Figure 2:**
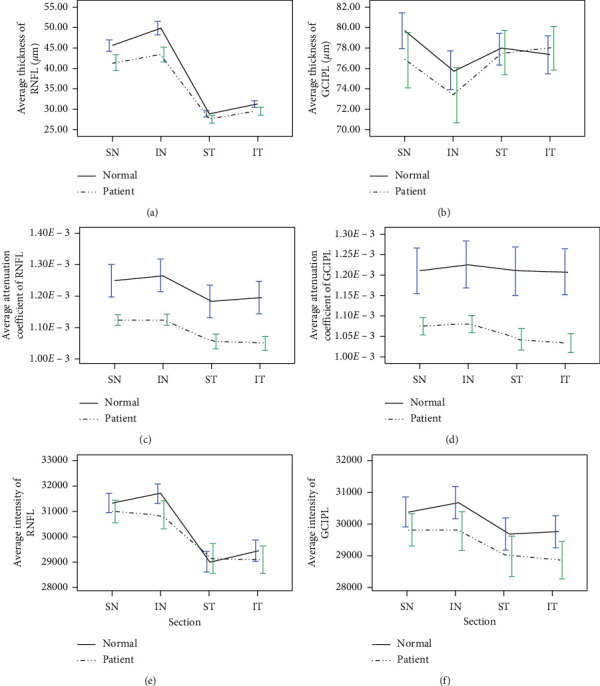
Comparison of thickness (*μ*m), attenuation coefficient (*μ*m^−1^), and corrected intensity (AU) in the RNFL and GCIPL between patients and controls according to each quadrant.

In patients, the overall mean thickness value of the RNFL was 35.44 ± 3.90 *μ*m, and this was lower compared to the thickness of controls (38.82 ± 3.07 *μ*m, *P* < 0.001). Significant differences existed between the groups for thickness measurements in all quadrants of the RNFL (Figure 2(a)), with the mean thickness reduced by about 9.64%, 13.02%, 4.67%, and 5.67% in SN quadrant (*P* < 0.001), IN quadrant (*P* < 0.001), ST quadrant (*P*=0.032), and IT (*P*=0.008) quadrant, respectively. The thickness values in the GCIPL of patients were lower compared with controls except those in the IT quadrant, but they were without statistical significance (*P*=0.596) (Figure 2(b)).

The mean attenuation coefficient for RNFL among patients was significantly lower compared to the control group in overall average value (*P* < 0.001), SN quadrant (*P* < 0.001), IN quadrant (*P* < 0.001), ST quadrant (*P*=0.001), and IT quadrant (*P* < 0.001) (Figure 2(c)); the corresponding values for GCIPL presented the same significant differences (Figure 2(d)).

The mean values of the corrected optical intensity were decreased in most quadrants of the interested layers in the patients compared with the controls, except in the ST quadrant of the RNFL. However, only the differences of values in IN quadrant of RNFL (*P*=0.006) and GCIPL (*P*=0.019), IT quadrant (*P*=0.022), and average of GCIPL (*P*=0.032) reached statistical significance (Figures 2(e) and 2(f)).

### 3.3. Associations of Attenuation Coefficient with Thickness and Corrected Intensity

**Figure 3:**
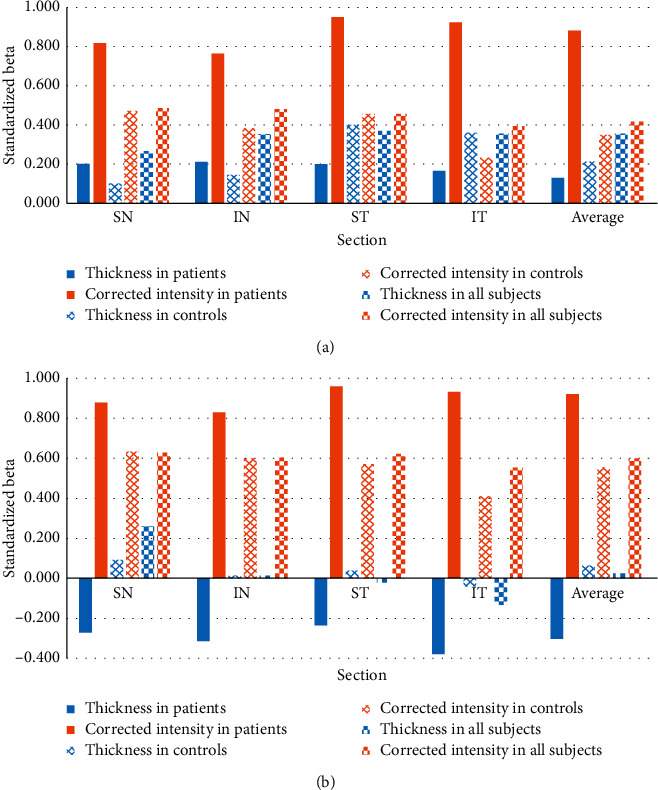
Correlations between attenuation coefficient and thickness and the corrected intensity in RNFL (a) and in GCIPL (b) for patients, controls, and all subjects, respectively.

In the linear regression analysis (Figure 3), the corrected intensities were strongly associated with the attenuation coefficients in all sections of RNFL and GCIPL (standardized beta's values within the range of 0.766 to 0.948 and 0.827 to 0.957, all *P* < 0.001) for patients. In controls, the correlations between these two indices were lower, but they were also significant.

Thickness presented weak correlations with the attenuation coefficients for all sections of RNFL in patients and controls, with the standardized beta's values within the range of 0.199 to 0.213 and 0.102 to 0.398 (most *P* > 0.05, except for *P*=0.008 in the ST quadrant and *P*=0.019 in the IT quadrant in controls). The corresponding correlations presented negative standardized beta's values in all quadrants of GCIPL among patients (ranged from −0.387 to −0.242) without significance except for the IT quadrant (*P*=0.016) (Figure 3(b)) and positive standardized beta's values in most quadrants of GCIPL (ranged from 0.004 to 0.088, except −0.038 in IT quadrant) in controls without significance.

**Figure 4:**
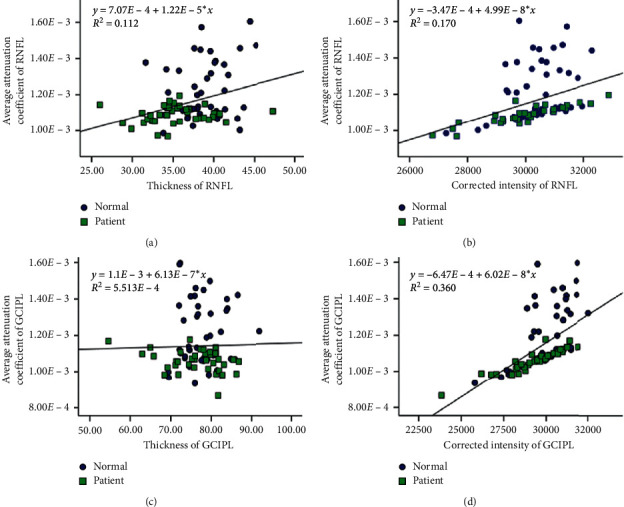
Linear regression analyses of the attenuation coefficient, the thickness, and the corrected intensity in the overall RNFL and GCIPL.

In all subjects, the average attenuation coefficients in RNFL and GCIPL significantly increased with the average corrected intensity in the corresponding layers (standardized beta = 0.413 and 0.600, all *P* < 0.001). The thickness of RNFL was significantly associated with the average attenuation coefficient (standardized beta = 0.335, *P*=0.002), while the thickness of GCIPL was not (Figures 3 and 4).

### 3.4. Diagnostic Performances of the Indices

**Figure 5:**
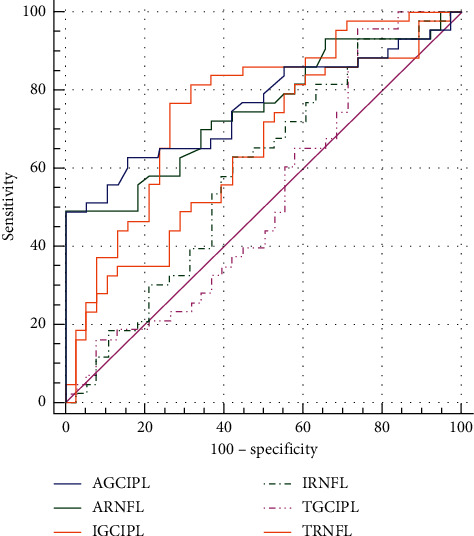
Receiver operating characteristic curves (ROC) of the thickness, the attenuation coefficient, and the corrected intensity index in the overall RNFL and GCIPL (expressed as TRNFL and TGCIPL, ARNFL and AGCIPL, and IRNFL and IGCIPL, respectively).

The ability of the index to differentiate between patient and control was investigated by comparing the AUC values. The mean thickness and attenuation coefficient in the RNFL presented better diagnostic accuracy than the corrected intensity (AUC value = 0.764, 0.751, and 0.589, *P* < 0.001, *P* < 0.001, and *P*=0.170). The AUC of the mean attenuation coefficient and corrected intensity in the GCIPL (AUC value = 0.758 and 0.639, *P* < 0.001, and *P*=0.032) was significantly higher than that of the thickness (AUC value = 0.523, *P*=0.726) for discriminating pituitary adenoma patients from normal controls (Figure 5 and Table 2). Pairwise comparisons of the AUC between the significant indices showed that the attenuation coefficient was significantly better than the corrected intensity in the diagnostic ability (*P*=0.004) in GCIPL, while the diagnostic ability of the thickness and the attenuation coefficient in RNFL is close (*P*=0.859) (Table 2).

## 4. Discussion

In this study, we conducted quantitative estimations of the attenuation coefficient, the corrected intensity, and the thickness of the RNFL and GCIPL from SD-OCT data. The retinal light reflection characteristics were debilitated in patients with pituitary adenoma. The results highlighted the potential of attenuation coefficients of the RNFL and GCIPL as the discriminant index for pituitary adenoma.

RNFL and GCIPL were selected as the layer of analysis because of their association with RGCs: the RNFL consists of ganglion cells axons, the GCL consists of ganglion cell bodies, and the IPL consists of ganglion cell dendrites [19]. The thickness of RNFL was significantly thinner in patients confirmed axonal injury due to the effect of pituitary adenoma to the optic chiasm [20, 21], particularly in the SN and IN quadrants (Table 1). However, the thickness of the corresponding quadrants in the GCIPL was not significantly thinned and even those values in the IT quadrant were higher compared between the patients and controls. These results indicated that the damage to the axon was greater than that of the bodies of RGCs, which are the first to suffer the damage. The injury will gradually penetrate to the bodies and dendrites of RGCs and aggravate as the damage to the optic chiasm increases, causing the dysfunction and/or apoptosis to occur in the RGCs.

The attenuation coefficients were significantly lower in RNFL and GCIPL among patients than those values among controls (Table 1 and Figure 2) and presented better ability to distinguish between patients and controls (Figure 5), which reflected the structural changes more. Compared with the thickness, the corrected intensity was less sensitive in the RNFL and more sensitive in the GCIPL in distinguishing pituitary adenoma from controls (Table 2 and Figure 5), with lower values in most quadrants of the RNFL and GCIPL of patients. The intensity and the attenuation coefficient are determined by the light reflective characteristics of the retinal layer tissues, which are affected not only by the retinal layer structure, including the number and distribution of cells, but also by the chemical gradient [21]. When RGC dysfunction emerges, even if it is not severe enough to cause the RGCs to die, the density of nerve fibers decreases and the RNFL and GCIPL have lower reflectivity, presenting lower corrected intensity and attenuation coefficient than that in controls. The attenuation coefficients in the RNFL and GCIPL significantly increased along with the increases of the corrected intensities, while those values were not significantly positively related to the thickness (Figure 4). These confirmed that retinal structural changes in RGCs did not necessarily cause thickness changes.

To compensate the weak raw signal resulting from the attenuation in retinal tissues, the optical depth compensation of OCT signal is necessary. The attenuation coefficient and the corrected intensity were estimated from the OCT data using a depth-dependent single scattering model in this study. Assuming that the same scattering model can be used for each sublayer, the depth-dependent single scattering model considers that a light wave is transmitted to the specimen to be imaged, which in turn backscatters a fraction of the incident light; the local attenuation of the propagating beam at depth *z* can be assumed to be proportional to the local reflectivity [16]. Each layer of the retina has the pixel-wise attenuation coefficient, rather than being considered homogeneous and having a uniform attenuation coefficient, as we did in the previous study [11]. The model has been validated to be effective in the attenuation compensation and contrast enhancement of OCT imaging artifacts on multilayer and heterogeneous tissues, just as the retina [12, 13, 16]. The attenuation coefficients and the corrected intensities obtained based on the model and the conclusions derived should be considered more comprehensive.

It should be pointed out that a more precise model considering the point spread function (PSF) of the catheter *P*(*z*) and the OCT signal depth-dependent sensitivity decay *S*(*z*) is also used in the attenuation correction of OCT data. *E*_*A*_(*z*) should be replaced by (*E*_*A*_(*z*)/*P*(*z*)*S*(*z*)); that is, the denoised SD-OCT signal should be corrected by PSF and the sensitivity roll-off. The attenuation coefficient $\hat{\mu }\left(z\right)$ deduced from the comprehensive model will be smaller than the value of *μ*(z) derived in this study, because the values of *P*(*z*) and *S*(*z*) range from 0 to 1 and decrease as *z* increases [22]. The sensitivity roll-off function and the confocal function have been studied and can be considered for more precise attenuation estimation [22]. This can be considered as a limitation in this study. However, the difference in retinal optical properties between controls and patients remained in this study because all SD-OCT data were processed by using the same method. In addition, multiple scattering-based OCT single model considering the second- and higher-order scattering processes have been studied in the past [23]; however, study has also shown that there is no significant difference between the single scattering model and the multiscattering model for the depiction of OCT signals [9].

Although the algorithm used in this study can enhance the contrast and compensate the attenuation, it still has some issues that need to be addressed in the application of clinical. The principal assumption of the signal scattering model based attenuation compensation method is that only light that has been backscattered once contributes to the OCT signal. Tissues such as the cornea, cristalline lens, and vitreous body are not opaque or highly scattered to the incident light, and OCT cannot image them well. In this algorithm, the area above the RNFL interface was regarded as the noise, and it did not participate in the attenuation coefficient calculation. Numerically, the attenuation coefficient is the ratio of the grayscale of the attenuated image to twice the sum of the image intensities of the entire retinal layers in the A-scan. A slightly larger value of the attenuation coefficient was obtained. A precise image segmentation algorithm was used to determine the upper interface of RNFL and lower choroid boundary in the preprocessing step. All of these need to be added into the image postprocessing software of the available OCT equipment, in order to achieve the automatic implementation of attenuation compensation to raw OCT data and attenuation coefficients derived.

## 5. Conclusions

In this paper, we have compared the macular retinal structural changes between patients with pituitary adenoma and controls using the attenuation coefficient, the corrected intensity, and the thickness derived from the SD-OCT scans. We discovered the changes in the retinal thickness and light reflection characteristics, as well as the effectiveness of the attenuation coefficients of RNFL and GCIPL in distinguishing patients from normal controls. The results indicated that the localized quantitative measurement of the attenuation coefficient could be used as an aided discriminant index and can significantly improve the clinical value of OCT.

## Figures and Tables

**Table 1:** Comparison of derived indices between patients and controls according to each quadrant and overall mean of interested layer.

Thickness			Attenuation coefficient			Corrected intensity		
Sections	Patient	Control	*P*	Patient	Control	*P*	Patient	Control	*P*
RNFL									
SN	41.32 ± 5.67	45.73 ± 4.61	**<0.001**	0.00112 ± 0.00005	0.00125 ± 0.00017	**<0.001**	31023.94 ± 1287.96	31358.52 ± 1215.93	0.233
IN	43.37 ± 5.63	49.86 ± 5.34	**<0.001**	0.00112 ± 0.00006	0.00127 ± 0.00017	**<0.001**	30877.51 ± 1636.81	31708.25 ± 1233.05	**0.006** ^*∗*^
ST	27.53 ± 3.02	28.88 ± 2.50	**0.032**	0.00106 ± 0.00007	0.00118 ± 0.00017	**0.001**	29176.80 ± 1759.36	29030.85 ± 1308.46	0.671
IT	29.47 ± 3.33	31.24 ± 2.55	**0.008**	0.00105 ± 0.00007	0.00120 ± 0.00017	**<0.001**	29110.76 ± 1598.85	29462.52 ± 1333.41	0.284
Average	35.44 ± 3.90	38.82 ± 3.07	**<0.001**	0.00109 ± 0.00005	0.00122 ± 0.00016	**<0.001**	30051.27 ± 1310.56	30381.26 ± 993.33	0.170^*∗*^
GCIPL									
SN	76.75 ± 8.21	79.56 ± 5.64	0.302^*∗*^	0.00108 ± 0.00006	0.00121 ± 0.00018	**<0.001**	29767.34 ± 1535.41	30326.60 ± 1519.34	0.167^*∗*^
IN	73.37 ± 8.15	75.76 ± 6.18	0.334^*∗*^	0.00108 ± 0.00006	0.00122 ± 0.00018	**<0.001**	29732.80 ± 1823.68	30609.55 ± 1619.45	**0.019** ^*∗*^
ST	77.47 ± 6.65	77.80 ± 4.98	0.803	0.00105 ± 0.00008	0.00121 ± 0.00019	**<0.001**	28940.98 ± 1935.87	29645.52 ± 1607.02	0.108^*∗*^
IT	77.90 ± 6.35	77.24 ± 6.01	0.596^*∗*^	0.00104 ± 0.00007	0.00121 ± 0.00019	**<0.001**	28821.85 ± 1752.33	29708.56 ± 1656.98	**0.022**
Average	76.37 ± 6.88	77.76 ± 4.87	0.726^*∗*^	0.00106 ± 0.00006	0.00121 ± 0.00018	**<0.001**	29320.48 ± 1593.57	30073.24 ± 1403.71	**0.032** ^*∗*^

RNFL, retinal nerve fiber layer; GCIPL, ganglion cells and inner plexiform layer; SN, superonasal; IN, inferonasal; ST, superotemporal; IT, inferotemporal. Unpaired *t*-test. ^*∗*^Mann–Whitney *U* test. Mean ± standard deviation. Bold numbers indicats significant statistical significance.

**Table 2:** Comparison of the areas under the receiver operating characteristic curves (AUCs) between the indices.

	RNFL			GCIPL		
	AUC (SE)	95% CI	*P*	AUC (SE)	95% CI	*P*
Thickness	0.764 (0.054)	0.659–0.870	**<0.001**	0.523 (0.066)	0.393–0.653	0.726
Attenuation coefficient	0.751 (0.054)	0.645–0.856	**<0.001**	0.758 (0.054)	0.652–0.864	**<0.001**
Corrected intensity	0.589 (0.064)	0.463–0.715	0.170	0.639 (0.062)	0.518–0.760	**0.032**
Difference between significant indices	0.013 (0.076)	−0.136–0.162	0.859	0.120 (0.042)	0.038–0.201	**0.004**

RNFL, retinal nerve fiber layer; GCIPL, ganglion cell and inner plexiform layer. CI, confidence interval. Bold numbers indicate significant statistical significance.

## Data Availability

Quantitative analysis data and 3D SD-OCT raw data are available on Figshare: https://doi.org/10.6084/m9.figshare.12645167.v1, https://doi.org/10.6084/m9.figshare.4964318.v1, and https://doi.org/10.6084/m9.figshare.4964216.v1.

## Supplementary Materials

Supplementary Materials Derivation of optical property indices.
