# Supplementary material for: Quantitative Analysis of Macular Retina Using Light Reflection Indices Derived from SD-OCT for Pituitary Adenoma
Source: J Ophthalmol. 2020 Nov 4;2020:8896114. doi: 10.1155/2020/8896114 (PMC7658689; doi:10.1155/2020/8896114)
Supplement: Supplementary Materials — Derivation of optical property indices. [file 8896114.f1.docx]

**Supplementary Materials**

**Derivation of optical property indices**

According to Lambert-Beer’s law, the irradiance of the incident light beam *E*(z) is given by Equation S1:

$E\left( z \right)=E_{0}e^{-\int_{0}^{z} \mu(u)du}$ (S1)

where *E*_0_ is the initial irradiance of the incident light beam at depth *z*=0, *μ*(z) (expressed in *μm*^-1^, *z* expressed in *μm*) is the depth-dependent attenuation coefficient. The denoised SD-OCT images were analyzed to quantify the attenuation coefficient *μ*(z) of the tissues by fitting the OCT signal to a depth-resolved single scattering model, defined as:

$E_{A}\left( z \right)=\alpha\cdot E_{0}\cdot r\left( z \right){\cdot e}^{-2\int_{0}^{z} \mu(u)du}$ (S2)

$E_{R}\left( z \right)=\alpha\cdot E_{0}\cdot r\left( z \right)$ (S3)

where *E_A_*(z) is the attenuated OCT A-scan signal, *α* is a possible conversion factor remaining that from the OCT postprocessing,$r\left( z \right)=\beta\cdot\mu(z)$ is the local reflectivity assuming a fixed ratio *β* to *μ*(z) at the depth *z*, *E_R_*(z) is the actual depth-reflectivity profile. By integrating Equation S2 on both sides, we have

$\mu\left( z \right)=\frac{E_{A}(z)}{2K\int_{z}^{\infty} E_{A}(u)du}$ (S4)

$E_{R}\left( z \right)=E_{A}(z)\frac{\int_{0}^{\infty} E_{A}(u)du}{\int_{z}^{\infty} E_{A}(u)du}$ (S5)

where $K=\alpha\beta E_{0}$ can be adjusted to tissue characteristics. By discretizing Equation S4 and Equation S5, the attenuation coefficient and the attenuation corrected A-scan are estimated as Equation S6 and Equation S7:

$\mu\left( z \right)\approx\frac{E_{A}(z)}{2K\sum_{u=z+1}^{N} E_{A}(u)}$ (S6)

$E_{R}\left( z \right)\approx E_{A}(z)\frac{\sum_{u=1}^{N} E_{A}(u)}{\sum_{u=z+1}^{N} E_{A}(u)}$ (S7)

where *N* is the final pixel in the A-scan. In Equations S6 and S7, the denominators depend on the depth information, the lower value of which means the higher attenuation of tissues, and the compensation factor is relatively higher to increase the local intensity.
